# Clinical and Immunological Features of Human *Leishmania (L.) infantum*-Infection, Novel Insights Honduras, Central America

**DOI:** 10.3390/pathogens9070554

**Published:** 2020-07-10

**Authors:** Wilfredo Sosa-Ochoa, Concepción Zúniga, Luis Fernando Chaves, Gabriela Venicia Araujo Flores, Carmen Maria Sandoval Pacheco, Vania Lúcia Ribeiro da Matta, Carlos Eduardo Pereira Corbett, Fernando Tobias Silveira, Marcia Dalastra Laurenti

**Affiliations:** 1 Laboratory of Pathology of Infectious Diseases, Medical School, São Paulo University, São Paulo 01246-903, SP, Brazil; wilfredo.ochoa@usp.br (W.S.-O.); gabyaraflo@usp.br (G.V.A.F.); carmenmsandovalp@usp.br (C.M.S.P.); mattav@usp.br (V.L.R.d.M.); ccorbett@usp.br (C.E.P.C.); 2 Microbiology Research Institute, Universidad Nacional Autónoma de Honduras, Tegucigalpa 11101, Honduras; 3 Department of Health Surveillance, University School Hospital, Tegucigalpa 11101, Honduras; concepcionzuniga@gmail.com; 4 Instituto Costarricense de Investigación y Enseñanza en Nutrición y Salud (INCIENSA), Tres Ríos 4-2250, Cartago, Costa Rica; lfchavs@gmail.com; 5 Parasitology Department, Evandro Chagas Institute (Surveillance Secretary of Health, Ministry of Health), Belém 66090-000, PA, Brazil; fernandotobias@iec.gov.br; 6 Tropical Medicine Institute, Pará Federal University, Belém 66075-110, PA, Brazil

**Keywords:** *Leishmania (L.) infantum*, human infection, cross-sectional study, prevalence, clinical-immunological profiles, Honduras

## Abstract

*Leishmania (Leishmania) infantum* is the etiological agent of both American visceral leishmaniasis (AVL) and non-ulcerated cutaneous leishmaniasis (NUCL) in Honduras. Although AVL is the most severe clinical form of infection, recent studies have shown that human immune response to parasite infection can result in a clinical-immunological spectrum. The overall prevalence rate of infection and clinical-immunological profiles of the *L. (L.) infantum* infection in Amapala municipality, South Honduras was determined. We examined 576 individuals with diagnosis based on combined ELISA (IgG/IgM) and DTH assays. We also used genus-specific kDNA PCR and Hsp70 PCR-RFLP for NUCL cases. Clinical evaluation found 82% asymptomatic and 18% symptomatic individuals. All symptomatic cases (n = 104) showing NUCL were positive for parasites. We identified *L. (L.) infantum* species in 100% of the skin lesion scrapings and in 90% of the blood samples from NUCL cases studied. A total of 320 asymptomatic individuals were exposed (ELISA+ and/or DTH+), providing an overall *L. (L.) infantum* prevalence of 73.6%. Clinical, parasitological, and immunological evaluations suggest seven infection profiles, three asymptomatic and four symptomatic. This represents the first report on clinical and immunological features of human *L. (L.) infantum*-infection in Amapala municipality, Honduras.

## 1. Introduction

Leishmaniases have a broad clinical and immunological spectrum of manifestations depending on *Leishmania* species, host immune responses, and possibly insect vector saliva factors [1]. In the New World, *Leishmania (Leishmania) infantum* causes subclinical manifestations and active visceral leishmaniasis (VL), also known as American visceral leishmaniasis (AVL), the latter being potentially fatal if untreated [2,3,4,5,6].

However, in Central America, *L. (L.) infantum*-infections have also shown atypical manifestations in humans, causing frequently non-ulcerated cutaneous leishmaniasis (NUCL) in adolescents and young adults, and sometimes causing AVL in children under ten years [7,8,9,10]. It is important to mention that both clinical forms are caused by genotypically similar parasites according to molecular tools [11]. NUCL is clinically characterized by non-ulcerated skin lesions, which most frequently are few and small in size, with chronic evolution, affecting the skin in the form of painless papules, nodules, and erythematous plaques surrounded by a hypopigmented halo [12,13].

Histopathological studies have shown that skin lesions of NUCL cases are characterized by a moderate mononuclear inflammatory infiltrate, predominantly formed by lymphocytes, with the presence of granulomas and scarce parasitism [13,14]. However, the co-occurrence of AVL and NUCL caused by *L. (L.) infantum* in the same geographic area suggests that host immunity factors may be involved in determining these different clinical forms of disease [15,16].

Here, we study the prevalence of human symptomatic and asymptomatic *L. (L.) infantum* infection in the population of Amapala municipality, Honduras. We use clinical, parasitological, and immunological diagnostics based on humoral (ELISA-IgG/IgM) and cellular (DTH skin test) immune responses. This combination of diagnostics renders possible the definition of a clinical-immunological infection spectrum in Central America. In this study, we followed a diagnostic protocol similar to the one used to study human *L. (L.) infantum*-infection in Amazonian Brazil, which described the evolution of clinical-immunological human infection profiles, two symptomatic (symptomatic infection (SI = AVL), oligosymptomatic infection (OSI)) and three asymptomatic (indeterminate initial infection (III), resistant subclinical infection (RSI), and asymptomatic infection (AI)), using results from cellular and humoral immune responses [17,18,19,20,21]. However, considering that, in Central America, *L. (L.) infantum*-infection is almost always dermal (NUCL), the aforementioned diagnostic approach, originally developed for AVL, allowed the identification of seven infection profiles in Amapala municipality, Honduras, three asymptomatic (indeterminate asymptomatic infection (IAI), resistant asymptomatic infection (RAI), and final asymptomatic infection (FAI)), and four symptomatic [indeterminate symptomatic infection (ISI), resistant symptomatic infection (RSI), final symptomatic infection (FSI), and early symptomatic infection (ESI)), which are also described in this study.

## 2. Results

### 2.1. Clinical and Demographic Aspects of Studied Subjects

A total of 576 individuals were evaluated from April to June 2017. From the evaluated individuals, 336 (58.3%) were female and 240 (41.7%) were male. Most individuals involved in the study (55.9%) were over 21 years old (Table 1). Skin lesions compatible with NUCL were identified in 104 individuals (18%), while 472 (82%) were apparently asymptomatic. The diagnostic was based on direct examination of Giemsa-stained smears from skin lesion scrapings from each individual, where amastigote forms of *Leishmania* sp. were observed in 100% of NUCL cases using a 100X objective in a light microscopy. The distribution of NUCL cases was not homogeneous regarding age (Table 1). In contrast, there was no difference in the proportion of cases by sex (Table 2). Most NUCL cases came from the northern part of the island, from San Pablo (24%) and Las Pelonas (10.6%) (Table 3). 

### 2.2. Parasitological and Molecular Diagnosis of Clinical Cases

**Figure 1:**
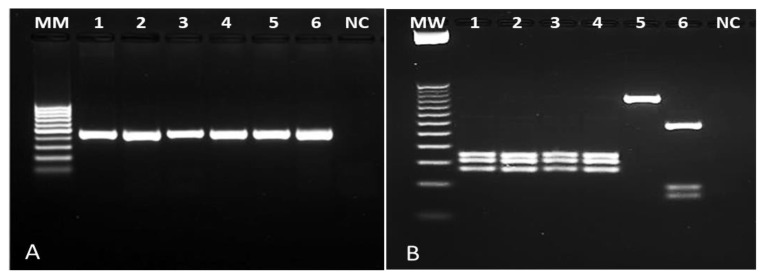
Photograph of agarose gel for samples of NUCL patients. (**A**): genus-specific PCR revealing a 120 bp fragment of *Leishmania* kDNA. (**B**): PCR-RFLP after *Hae* III digestion of a 234 bp Hsp70 fragment. Columns 1–3: lesion scraping samples from NUCL patients; columns 4-6: *Leishmania (L.) infantum* (MHOM/BR/1972/BH46); *Leishmania (L.) amazonensis* (MHOM/BR/1973/M2269), *Leishmania (V.) braziliensis* (MHOM/BR/1995/M15280), respectively. NC: negative control; MW: ladder.

Of the 576 individuals evaluated, 104 (18%) had skin lesions compatible with NUCL. Amastigote forms of *Leishmania* sp. were observed in 100% of these cases by Giemsa staining. To confirm *Leishmania* infection, 30 whole blood samples and 45 skin lesion scrapings (all with positive microscopy) were submitted to a genus-PCR targeting *Leishmania* kDNA and further to the species identification by PCR-RFLPs through *Hae* III digestion of a Hsp70 fragment. All samples analyzed were positive for *Leishmania* sp. Respective to the characterization, Hsp70-RFLP identified *L.* (*L.*) *infantum* in 90% (27/30) of the blood samples and in 100% of the skin lesion scrapings (45/45), allowing to confirm *L.* (*L.*) *infantum* as the pathogen causing NUCL lesions in Amapala municipality, Honduras (Figure 1).

### 2.3. Clinical Characteristics of NUCL Skin Lesions

Regarding the clinical characteristics of patients with NUCL, we found that most patients (63%/65) had only one lesion (Table 4). The majority of lesions were observed in the extremities and the chest (55% and 32%, respectively). The size of the skin lesion was bigger than 5 mm in 65% of the patients, and 40% (n = 42) of them had an evolution <12 months. There was no significant difference (Kruskal–Wallis test, df = 18; P = 0.8920) between the time of disease progression (months) and the number of skin lesions.

### 2.4. Prevalence rate of Human L. (L.) infantum-Infection in the Municipality of Amapala, Honduras

Human *L. (L.) infantum*-infection at our study site was also studied with humoral and cellular immunological tests (ELISA-IgM/IgG and DTH). Thus, considering that all symptomatic infections (NUCL) were confirmed by parasitological diagnostics (104), the prevalence rate of symptomatic infection was 18%, while among asymptomatic individuals examined (472), 320 were at least exposed according to results from the immunological tests (ELISA-IgM/IgG and DTH), generating an asymptomatic prevalence of 55.5%. When symptomatic (104) and asymptomatic infections (320) are added, a total of 424 infections increased the prevalence to 73.6% (Table 5).

### 2.5. Reactivity to DTH/ELISA in Asymptomatic and Symptomatic (NUCL) Individuals

None of the recruited individuals presented any clinical signs of AVL during our study period. Among the 472 individuals in the clinically asymptomatic group, 320 were identified with reactivity either for ELISA (IgG+/IgM+), for DTH+, or for both immunological tests simultaneously, which confirms the immunological diagnosis of infection in 67.8% of the asymptomatic individuals.

We were also able to characterize, among the 320 individuals with asymptomatic infections, three clinical-immunological profiles, namely: (1) indeterminate asymptomatic infection (IAI), characterized by a humoral immune response (ELISA: IgM+/IgG+), however, without detection of cellular immune response (DTH−) IgG+/IgM+/DTH−, IgG+/IgM−/DTH−, and IgG−/IgM+/DTH−; (2) resistant asymptomatic infection (RAI), characterized by humoral (ELISA: IgM+/IgG+) and cellular (DTH+) immune responses IgG+/IgM+/DTH+, IgG+/IgM−/DTH+, and IgG−/IgM+/DTH+; and, still, (3) final asymptomatic infection (FAI), characterized only by detection of cellular immune response (DTH+) IgG−/IgM−/DTH+.

On the other hand, in the 104 individuals with symptomatic infection (NUCL) with positive parasitological diagnosis, it was also possible to identify three similar clinical-immunological profiles, that is: (1) indeterminate symptomatic infection (ISI), characterized by humoral immune response (ELISA: IgM+/IgG+), however, without detection of cellular immune response (DTH−) IgG+/IgM+/DTH−, IgG+/IgM−/DTH−, and IgG−/IgM+/DTH−; (2) resistant symptomatic infection (RSI), characterized by humoral (ELISA: IgM+/IgG+) and cellular (DTH+) immune responses IgG+/IgM+/DTH+, IgG+/IgM-/DTH+, and IgG−/IgM+/DTH+; and, (3) final symptomatic infection (FSI), also characterized only by cellular immune response (DTH+) IgG−/IgM−/DTH+. In addition to these three profiles, it was also possible to identify a fourth clinical-immunological profile: (4) early symptomatic infection (ESI), in which only the parasitological diagnosis was present, while humoral (ELISA: IgM−/IgG−) and cellular (DTH−) immune responses were not detected.

In summary, considering the three type of diagnostic tests used in this study, clinical, parasitological, and immunological (ELISA-IgG/IgM and DTH), it seems clear that, depending on the individual’s immune response, that is, resistance or susceptibility, the infection can evolve to an asymptomatic resistance profile, as did most cases of the infection diagnosed here (75.5%; 320/424) without clinical signs of either NUCL or AVL, or to a symptomatic one, showing clinical signs of NUCL (24.5%; 104/424).

### 2.6. Polytomous Logistic Regression

Polytomous logistic regression showed that individuals who had a DTH+ test or combination with an early marker (DTH+/IgM+) had a greater chance of having characteristic NUCL lesions (DTH+ P = 0.001; DTH+/IgM+ P = 0.002). In both cases, the odds of NUCL were increased at least 2.8 times. This association between DTH+ and NUCL is an interesting feature of *L. (L.) infantum* infections in Central America. Nevertheless, individuals with only IgM+ test have 29% lower odds of NUCL according to the polytomous logistic regression (*p* < 0.007, Table 5), and the odds are even smaller for cases that are only IgG+, with 88.3% lower odds of presenting NUCL (*p* < 0.007, Table 5). The combination IgG−/IgM−/DTH− was taken as the reference group. Other results for the polytomous logistic regression are shown in Table 6.

## 3. Discussion

To the best of our knowledge, this is the first study about clinical-immunological profiles of the human *L. (L.) infantum*-infection spectrum in Mesoamerica. We combined clinical and parasitological examination as well as humoral (ELISA-IgM/IgG) and cellular (DTH skin test) immune response as parameters to understand the risk of individuals to get infection by *L. (L.) infantum* in an area where this pathogen is present [9,22,23].

Amapala municipality has several characteristics that enable human *L. (L.) infantum*-infection transmission [22,24]. For example, *Lutzomyia longipalpis* [25] and *Lutzomyia evansi* (Nuñez-Tovar), the two most important sand fly vector species in the region, are also the two most abundant species in tropical forests surrounding the study area [26]. These conditions for *L. (L.) infantum* endemicity are crucial to understand its clinical form prevalence patterns, since it has been suggested that a long-term exposure to *L. (L.) infantum*-infection may favor the development of some population level herd immunity, as reported in northeastern Brazil [17,19]. It has been shown that resistance against AVL could be controlled by a genetic mechanism related with the ability to mount an acquired immune response to *L. (L.) infantum*-infection as measured by the DTH^+^ phenotype associated with LECT2 and TGFBI genes [27,28]. Previous studies reported similar characteristics in NUCL patients from Honduras [22] and Nicaragua [8], describing positive DTH as the most relevant finding, 58% and 79%, respectively. Similar findings are reported in the present study that showed positive DTH reaction in both resistant clinical-immunological profiles, symptomatic (RSI and FSI) and asymptomatic (RAI and FAI), with rates of 55.7% and 32.4%, respectively (Table 5).

Regarding the gender distribution of human *L. (L.) infantum*-infections, no significant difference was found by sex (P > 0.82), as reported in preceding studies [17,22,29]; however, infections became more common as individuals aged. The macroscopic lesions were mostly unique, small (<5 mm), nodular, and not ulcerated with a hypopigmented halo, as previously described [8,22,30]. Lesions were mainly present in the extremities, followed by the thorax, with a time of evolution below 12 months.

Concerning the prevalence of infection in the study area, it is important to clarify that, considering the clinical criteria demonstrated by relevant participation in the diagnosis of symptomatic cases of infection (NUCL), since 100% of them were confirmed by parasitological examination, there is no doubt that the clinical parameter cannot be neglected when assessing the prevalence of infection, especially in the clinically suspected NUCL cases. Therefore, the prevalence of infection was first estimated considering the symptomatic cases (NUCL) confirmed by the parasitological exam (n = 104), which generated a prevalence rate of 18%, and, in the second, considering the cases of asymptomatic infection confirmed by immunological tests (ELISA-IgM/IgG and DTH), which revealed an asymptomatic prevalence rate of 55.5%. Finally, considering the numbers of cases from symptomatic (n = 104) with those asymptomatic (n = 320), it then reached a total of 424 cases of infection or exposure, revealing an overall prevalence rate of 73.6%.

There is no doubt that this reflects a very interesting epidemiological situation for the following reasons: first, because it highlights the importance of clinical suspicion in assessing the prevalence of symptomatic infection, since 100% of suspected NUCL cases were confirmed by parasitological examination; second, if a projection of NUCL is made in the “Del Tigre” island population (~5000 individuals) based on the determined symptomatic infection prevalence rate (18%), it is possible to estimate the existence of approximately 900 NUCL cases on the island, which represents important information for the epidemiological surveillance secretary of the Health Department of Amapala municipality; and third, although the prevalence of asymptomatic infection (55.5%) does not reveal an estimate of the disease itself, this estimate is of great value for better understanding the magnitude of the epidemiological situation of infection in the study area, where the overall prevalence rate of infection, which resulted from the prevalence of symptomatic infection plus the asymptomatic one, reached a very high rate of 73.6%, indicating that almost three thirds (75%) of the population in the study area are exposed or infected by *L. (L.) infantum*.

The importance of DTH (cellular immunity) as a diagnostic tool for human infection by viscerotropic *Leishmania* species has undoubtedly been largely demonstrated not only in the New World [8,22,27,31,32,33] but also in the Old World [34,35,36]. However, as shown in Table 5, the use of ELISA-IgM/IgG serological test (humoral immunity) cannot be neglected as an important tool in the diagnosis of infection, since, comparing the two diagnostic tools in the present study, it was verified that, on the spectrum of asymptomatic infection, the serological diagnosis (ELISA-IgM/IgG) of cases from the IAI profile was confirmed in 35.4% of the cases, while the diagnosis by cellular immunity test (DTH) of the cases of the RAI and the FAI profiles was confirmed in 32.4% of the cases, showing that there was no difference between these rates (P > 0.05) and that the two types of immunity, humoral and cellular, seem to be strongly associated in the course of asymptomatic infection. It is interesting to note, however, that, on the spectrum of symptomatic infection (NUCL), it was observed that the diagnosis by cellular immunity test (DTH) of cases from RSI and FSI profiles showed positivity in 55.7% of these cases, which is significantly higher (P < 0.05) than the 19.2% positive diagnoses among the cases from ISI profile, suggesting that, unlike the course of asymptomatic infection, on the side of the symptomatic infection spectrum (NUCL), the cellular immunity overlapped the humoral one, which possibly may be interpreted as an attempt to play a protective role against infection.

These results seem to definitively demonstrate that the diagnostic approach to human *L. (L.) infantum*-infection as well as the immune responses of the infected individuals in an endemic area should not be individualized in just one of the investigation methods, DTH (cellular immunity) or ELISA-IgM/IgG (humoral immunity), due the magnitude of human’s immune responses repertory against infection. In this sense, it is important to emphasize that the diagnostic approach to infection used in the present study was based on a diagnostic approach protocol that also sought to contemplate the diagnosis of the two types of immune responses of infection, cellular and humoral, in the Brazilian Amazon [17,18,19,20,21], which provided, in the present study as well as in the Amazonian studies, a greater visibility of the clinical-immunological profiles of infection spectrum with the identification of seven clinical-immunological profiles, three on the side of the asymptomatic infection spectrum (IAI, RAI, and FAI), and four on the side of the symptomatic infection (NUCL) spectrum (ESI, ISI, RSI, and FSI).

Regarding the importance of these clinical-immunological profiles of human *L. (L.) infantum*-infection in the study area, defined by the association of the clinical status of the individuals examined (symptomatic and asymptomatic), parasitological examination, and immunological tests (ELISA-IgM/IgG and DTH), it is worth mentioning that, among those on the side of the symptomatic infection (NUCL) spectrum, the clinical-immunological profile ESI mainly draws attention due to its clinically symptomatic infectious state confirmed by parasitological diagnosis, however, without detection of both humoral (ELISA-IgM/IgG) and cellular (DTH) immune responses. At first sight, this is a condition that seems to represent an early infectious state, reason that it was named early symptomatic infection; probably, there should not have been enough time for the parasitic load on the skin lesions of these patients to trigger an antigenic stimulus capable of promoting the full activation of humoral and/or cellular immune responses. In this sense, it is important to say that, in some NUCL cases observed in the present study, there were a very limited number of skin lesions (one or two only), papular in appearance (≤5 mm), and with a low parasitic load under microscopy, confirming the low parasitic load on the skin. Moreover, it is necessary to emphasize that this is a condition that seems unprecedented in the study of leishmaniases, since, to date, no other clinical-immunological situation regarding human symptomatic *Leishmania*-infection is known that is not supported by humoral (ELISA-IgM/IgG) or cellular (DTH) immune responses. In this way, the other explanation for this unusual fact could be the result of an escape mechanism of the parasite to humoral and/or cellular immune responses, which has already been evidenced in the Brazilian Amazon, mainly with regard to cutaneous leishmaniasis by *Leishmania (L.) amazonensis*, which uses this mechanism of escape from cellular immune response to promote the clinical forms borderline disseminated cutaneous leishmaniasis (LCDB) and anergic diffuse cutaneous leishmaniasis (LCAD), which are associated with important cellular immune-suppression (negative DTH response) and poor therapeutic response for different schemes of treatment [37,38,39].

Another point that deserves to be highlighted regarding the ESI profile is its high frequency (25%) among the symptomatic infection profiles, being surpassed only by the frequency (30.8%) of the FSI profile, which denotes that, among the clinical-immunological profiles of symptomatic infection (NUCL), the ESI profile may also signal a state of adaptation of the parasite on the host skin, possibly seeking to produce an immune-inflammatory response of moderate intensity with the objective of using the host as a source of infection for the sand fly vector (*Lutzomyia longipalpis*). In this sense, there is already evidence obtained by our group pointing to the moderate presence of inflammatory response [13] as well as mediators of the immune-inflammatory response in the skin lesions of patients with NUCL [14].

In spite of the crucial role of host immunity in determining the human humoral and cellular immune responses against *Leishmania* parasite, the genetic diversity of strains could not be neglected. In this sense, it is important to mention that homogeneity among *L. (L.) infantum* strains isolated from human VL and NUCL in Honduras has been described [11]. In addition, recent microsatellite studies have shown low heterogeneity among *L. (L.) infantum* isolates from the New World and no correlation between *L. infantum* genotypes and clinical picture [40]. Despite the description of MON-1 and non-MON-1 populations on the Caribbean Coast of Central America, it is important to mention that our study area is on the Pacific Coast of Honduras, where has been described the presence of MON-1 population. Genome-wide global study showed little diversity among *L. infantum* samples; however, several hybrid lineages were reported with identical genetic groups varying in heterozygosity and levels of linkage [41], which could be related to different host responses to infection. 

In the polytomous regression analysis, the presence or the absence of typical NUCL lesions was used as a response variable. Odd ratios were then estimated for different combinations of test results, and it was observed that coefficients for the group DTH^+^ (P < 0.002) and IgM^+^/DTH^+^ (P < 0.001) were the best predictors for having an NUCL lesion. These results help establish a possible interaction among exposure to *L. (L.) infantum*, the appearance of an early marker (IgM) and a resistance marker (DTH) in the development of immune protection in the inhabitants of the Pacific zone from Honduras. This represents the first report on clinical and immunological features of symptomatic and asymptomatic human *L. (L.) infantum*-infection in the municipality of Amapala, Pacific coast of Honduras.

## 4. Materials and Methods 

### 4.1. Study Area

This study was carried out in Amapala municipality (N13° 15.618, W87° 37.463), Department of Valle, with an area of 80.7 km^2^. The municipality comprises two islands, Zacate Grande and El Tigre, located in the Gulf of Fonseca in south-western Honduras. The municipality has a total of 28 villages with an estimated population of 13,302 individuals. The natural vegetation cover is dominated by a dry tropical forest. Temperature ranges between 25 °C and 35 °C, and average annual rainfall is 2096 mm. The dominant topography is mountainous and rugged with an average altitude of 44 m. 

However, the highest point of the island is 760 meters with clay soil, marshes, and swamps [42] The ecological landscape markedly changes between the dry season (November–May) and the rainy season (June–October). Housing characteristics in Amapala are homogeneous; most houses are made of adobe and/or wood with a tin roof and have electricity but lack sanitation. Most homes have at least one pet, with dogs being the most common pet [26,42].

### 4.2. Study Design

**Figure 2:**
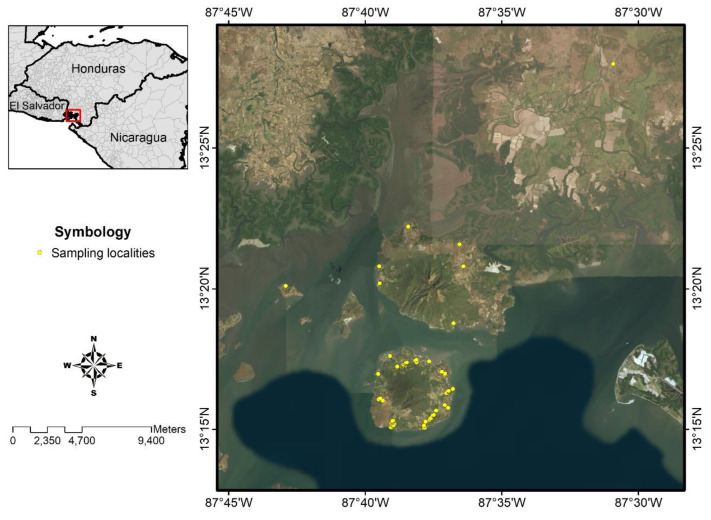
Study area, municipality of Amapala, Honduras. For details about the symbols, please refer to the inset legend.

A cross-sectional study was designed to study the prevalence of human symptomatic and asymptomatic *L. (L.) infantum*-infection using clinical and parasitological diagnostics as well as humoral (ELISA-IgG/IgM) and cellular (DTH skin test) immune responses, respectively, which also were used to clarify the clinical-immunological profiles of the infection spectrum. In all cases, we used *L. (L.) infantum*-specific antigens [19,20,43]. To calculate the sample size, we used WinEpiscope statistical program [44], which estimated that the population involved in the study should consist of a cohort of approximately 586 subjects aged 1 year or older, assuming an infection rate of 50%, since there are no previous studies carried out in Central America. We were able to 576 individuals from 15 locations in the municipality of Amapala, Department of Valle, Honduras (Figure 2).

### 4.3. Study Population

The individuals were invited to participate in the project in coordination with the Health Unit director of Amapala municipality. The sampling was done by active search, house by house, to aggregate a larger number of individuals. The study population consisted of 576 individuals, 336 females and 240 males older than one year. The population was divided into three groups according to age group: 1–10, 11–20, and ≥21 years.

The purpose of the study was presented to each individual, and only those who agreed to participate were included in the study by signing an informed consent form. The project was approved by the Ethics Committee of the Master Program in Infectious and Zoonotic Diseases of the School of Microbiology of the National Autonomous University of Honduras (protocol number 03-2014) and by the Research Ethics Committee of the School of Medicine of the University of São Paulo (CAAE protocol: 64223917.1.0000.0065).

### 4.4. Clinical Evaluation and Sample Collection

All enrolled subjects underwent a clinical evaluation. The clinical evaluation considered clinical signs suggestive of AVL and NUCL. Specifically, clinical signs included fever, liver and spleen enlargement, as well as papular or nodular skin lesions. Clinical and epidemiological data were recorded for each individual and used for further correlation with ELISA (IgM/IgG) and DTH findings. Individuals with skin lesions suspected of NUCL underwent direct parasitological examination using Giemsa-stained skin lesion scrapings and 100x objective light microscopy. Subsequently, the Montenegro skin test (DTH) was applied and whole blood collected, 5 mL, by venipuncture to obtain serum, which was aliquoted and stored at −70 °C until processing. 

### 4.5. Montenegro Skin Test—Delayed Type Hypersensitivity (DTH)

Promastigote forms of *L.* (*L.*) *infantum* (MCAO/BR/2003/M22697) were cultured in RPMI 1640 medium (Sigma-Aldrich, St. Louis, MO, USA). The parasites in stationary phase of growth in culture were washed and fixed in merthiolate solution (1/10,000) in the final concentration of 10^7^ forms/mL and stored in 2 mL bottles in refrigerator. A volume of 0.1 mL of antigen suspension was intradermally administered to the forearm of each individual. After 48 hours, the intradermal reaction was evaluated, and the formation of a nodule greater than or equal to 5 mm in diameter was considered positive. Equal volume of 1/10,000 merthiolate solution without *Leishmania* antigen was intradermally administered to the contralateral forearm of each individual as negative control [17,19,20,43].

### 4.6. Enzyme Linked Immune Sorbent Assay (ELISA)

ELISA was done according to Hirata and collaborators [45]. Polystyrene plates were sensitized with 100 µL of crude *L.* (*L.*) *infantum* antigen (MHOM/HN/2017/AMA-73) at a concentration of 5 µg/well diluted in carbonate-bicarbonate buffer pH 9.6. The plates were incubated overnight at 4 °C. Following 3 washes with Tween-20 phosphate-buffered saline (PBS), nonspecific binding sites were blocked with 100 µL 10% skim powdered milk solution in PBS for 1 hour at 37 °C in a humid chamber. After washing the plates, duplicate test sera and controls diluted 1:400 in Tween-20 PBS were added in the volume of 100 µL per well. After incubation at 37 °C for 1 hour, the plates were washed again, and the anti-human IgG peroxide conjugate (Calbiochem) at dilution of 1:50,000 or the anti-human IgM peroxide conjugate (Calbiochem) at dilution of 1:20,000 was added in 100 µL/well volume, and the plates were incubated at 37 °C for 45 minutes. After washing the plates, the reaction was finalized by the addition of 100 µL/well of 3,3′, 5,5′ tetramethylbenzidine (TMB) substrate (B&D) and kept for 15 minutes at room temperature in a darkroom. The reaction was stopped with 50 µL/well of 2N sulfuric acid, and the reading was performed on a spectrophotometer on a 450 nm filter. The observed absorbance values were corrected by subtracting the blank absorbance value. To establish the reaction cut-off line, the mean absorbance values obtained for negative control sera were increased twice by their standard deviation. Samples with absorbance values lower than the reaction cut-off value were considered negative, and samples with values above the cut-off value were considered positive. Considering the optical density values of the positive control and the tests, the absorbance of the positive samples were converted to ELISA units according to Rodriguez-Cortez et al. [46]

### 4.7. Identification Criteria of Human L. (L.) infantum-Infection

The definition of human *L.* (*L.*) *infantum*-infection or exposure was determined by clinical examination, parasitological diagnosis, and the presence of reactivity in one or both immunoassays (ELISA-IgM/IgG and DTH).

### 4.8. Molecular Diagnosis and Characterization of Leishmania Parasite Species

A commercial kit (Wizard® Genomic DNA Purification Kit, Promega, USA) was used for DNA extraction from blood samples following the manufacturer’s instructions. DNA from skin lesion scrapes samples was extracted using Chelex 5% (Bio-Rad Lab Inc., Hercules, USA).

To identify the *Leishmania* genus, primers LEISH-1: 5′-AACTTTTCTGGTCCTCCGGGTAG-3′ and LEISH-2: 5′-ACCCCCAGTTTCCCGCC-3′ were used that provided a kDNA product of 120 bp [47]. Amplifications were performed using a commercial kit (Master Mix 2X -Promega). Each reaction was performed by adding 4 µL of target DNA and 0.6 µmol/L of each primer in a final volume of 20 µL. The amplifications consisted of an initial denaturation cycle at 94 °C for 5 min. The second step of 40 cycles consisted of denaturation at 95 °C for 15 s, annealing at 60 °C for 20 s, and extension at 72 °C for 1 min, followed by a final extension cycle at 72 °C for 10 min. Results were observed on a 2% agarose gel, previously subjected to a 100 V electrophoresis for 1 h. 

In order to characterize *Leishmania* species, a PCR-RFLP targeting a fragment of 234 bp of Hsp70 gene was accomplished with primers Hsp70 sense (5′ GGACGAGATCGAGCGCATGGT 3′) and Hsp70 antisense (5′ TCCTTCGACGCCTCCTGGTTG 3′) [48]. Amplifications were performed using a commercial kit (Master Mix 2X -Promega). Each reaction comprised 4 µL of sample DNA and 0.6 µmol/L of each primer in a final volume of 20 µL. The amplifications consisted of an initial denaturation cycle at 94 °C for 5 min. The second step consisted of denaturation at 94 °C for 30 s, annealing at 59 °C for 1 min, and extension at 72 °C for 1 min for 37 cycles. The last step consisted of a final extension cycle at 72 °C for 10 min. Results were observed on a 2% agarose gel previously subjected to a 100 V electrophoresis for 1 h. To perform the restriction of PCR products, 15 µL of the amplified DNA were added to a tube reaction containing 1 µL (10 U) of *Hae* III enzyme (Promega), 2 µL of restriction enzyme 10X buffer, 0.2 µL of acetylated BSA at 10 µg/µL and 1.8 µL of deionized water, incubated at 37 °C for 3 h followed by 20 min incubation at 80 °C for the enzyme inactivation. The species profiles of each sample and reference controls were observed in a 4% agarose gel subjected to electrophoresis for 3.5 h.

### 4.9. Data Analysis

The data were analyzed using Stata statistical package V.12.0 (StataCorp, 2011. College Station, TX: StataCorp LP) and WinEpisocope 2.0. The Chi-Square Test of Independence was used to test for significant differences in infection between sex and age of symptomatic and asymptomatic groups with a 5% significance level. The Kruskal–Wallis test was used to estimate the median difference among the time of disease progression (months) and the number of skin lesions. We also performed a polytomous logistic regression to understand how the positivity of one or a combination of diagnostic tests was associated with NUCL as a clinical outcome [49].

## 5. Conclusions

The combination of clinical, parasitological, and immunological (ELISA-IgM/IgG and DTH) parameters allowed us to estimate *L.* (*L.*) *infantum* prevalence and to characterize the clinical-immunological profiles of its infection spectrum at Amapala municipality in Honduras. We found that skin symptomatic infections, called non-ulcerated or atypical cutaneous leishmaniasis, were associated with a positive DTH response and, to a lesser extent, with IgM/IgG production. 

## Figures and Tables

**Table 1:** Demographic age profile of the individuals enrolled in the study of *Leishmania (L.) infantum*, Municipality of Amapala, Honduras, 2017.

Characteristics	Patients with NUCL (n = 104)		Patients without NUCL (n = 472)		Total n = (576)		
	n	(%)	n	(%)	n	(%)	
**Age (years)**							
1–10	21	20.19	38	8.05	59	10.24	
11–20	44	42.31	151	31.99	195	33.85	* χ2 = 20.30; df = 1; P = 0.000
≥21	39	37.50	283	59.96	322	55.90	

* Comparison between patients under 21 with and without injuries. NUCL: non-ulcerated cutaneous leishmaniasis.

**Table 2:** Demographic profile by sex of the individuals enrolled in the study of *Leishmania (L.) infantum*, Municipality of Amapala, Honduras, 2017.

Characteristics	Patients with NUCL (n = 104)		Patients without NUCL (n = 472)		Total n = (576)		
	n	(%)	n	(%)	n	(%)	
**Sex**							
F	69	66.35	267	56.57	336	58.33	** χ2 = 3.03; df = 1; P = 0.082
M	35	33.65	205	43.43	240	41.67	

** Comparison between sex with and without injuries.

**Table 3:** Distribution of enrolled individuals by location in the study of *Leishmania (L.) infantum*, Municipality of Amapala, Honduras, 2017.

Characteristics		Patients with NUCL (n = 104)		Patients without NUCL (n = 472)		Total n = (576)	
		n	(%)	n	(%)	n	(%)
Locality	Geographical Coordinates						
Amapala Centro	N13.293356; W87.651540	8	7.69	10	2.12	18	3.13
San Pablo	N13.289431; W87.641807	25	24.04	96	20.34	121	21.01
Islitas	N13.260960; W87.623410	4	3.85	59	12.50	63	10.94
Las Pelonas	N13.282870; W87.618000	11	10.58	29	6.14	40	6.94
Tiguolotada	N13.262700; W87.615980	8	7.69	20	4.24	28	4.86
Playa Negra	N13.253710; W87.650100	6	5.77	68	14.41	74	12.85
Los Langues	N13.359536; W87.609056	9	8.65	21	4.45	30	5.21
Puerto Grande	N13.370042; W87.640430	4	3.85	21	4.45	25	4.34
Caracol	N13.282807; W87.658781	3	2.88	92	19.49	95	16.49
San Carlos	N13.334952; W87.715220	2	1.92	1	0.21	3	0.52
Playa Grande	N13.266940; W87.655970	11	10.58	6	1.27	17	2.95
Punta Onda	N13.272660; W87.615800	11	10.58	48	10.17	59	10.24
San Lorenzo	N13.366667; W87.266667	0	0.00	1	0.21	1	0.17
Campo Sol	N13.293356; W87.651540	1	0.96	0	0.00	1	0.17
Guarolita	N13.290137; W87.627655	1	0.96	0	0.00	1	0.17

**Table 4:** Clinical characteristics of symptomatic NUCL, Municipality of Amapala, Honduras.

Characteristics	NUCL	
	(n)	(%)
**Number of lesions**		
1	65	63
2–3	28	27
4–6	8	8
>6	3	3
Location (n = 207)		
Face	26	13
Extremity	114	55
Chest	67	32
Size (mm) (n = 207)		
<5	134	65
>5	73	35
Evolution (months)		
<12	42	40
12–23	23	22
24–47	14	14
>48	5	5
Did not remember	20	19

**Table 5:** Reactivity to DTH and ELISA-IgG/IgM in clinically asymptomatic and symptomatic (NUCL) human *L. (L.) infantum*-infections, Amapala municipality, Honduras, 2017.

	Asymptomatic n = 472 (%)	Symptomatic (NUCL) n = 104 (%)
IgG+/IgM+/DTH+	18 (3.8)	2 (1.9)
IgG+/IgM-/DTH+	28 (5.9)	4 (3.8)
IgG−/IgM+/DTH+	41 (8.7)	20 (19.2)
IgG−/IgM-/DTH+	66 (14.0)	32 (30.8)
IgG+/IgM+/DTH−	26 (5.5)	2 (1.9)
IgG+/IgM−/DTH−	33 (7.0)	2 (1.9)
IgG−/IgM+/DTH−	108 (22.9)	16 (15.4)
IgG−/IgM−/DTH−	152 (32.2)	26 (25.0)

**Table 6:** Results of the multivariable polytomus logistic regression analysis.

Predictor	Outcome	Odds Ratio	Std. Err.	Z	P>|z|	[95% Conf. Interval]	
**IgM−IgG−/DTH−**	NUCL −			Reference			
**IgM+**	NUCL +	0.866	0.295	−0.42	0.674	0.443	1.692
	NUCL −	0.710	0.089	−2.72	0.007	0.555	0.909
**IgM+/IgG+**	NUCL +	0.449	0.343	−1.05	0.295	0.100	2.009
	NUCL −	0.171	0.036	−8.32	0.000	0.112	0.259
**IgG+**	NUCL +	0.354	0.268	−1.37	0.171	0.080	1.566
	NUCL −	0.217	0.041	−7.95	0.000	0.149	0.316
**IgG+/DTH+**	NUCL +	0.835	0.480	−0.31	0.754	0.270	2.578
	NUCL −	0.184	0.037	−8.23	0.000	0.123	0.275
**DTH+**	NUCL +	2.834	0.857	3.45	0.001	1.567	5.127
	NUCL −	0.434	0.064	−5.66	0.000	0.325	0.579
**IgM+/IgG+/DTH+**	NUCL +	0.649	0.503	−0.56	0.578	0.142	2.966
	NUCL −	0.118	0.029	−8.56	0.000	0.072	0.193
**IgM+/DTH+**	NUCL +	2.852	0.985	3.03	0.002	1.448	5.614
	NUCL −	0.269	0.047	−7.45	0.000	0.191	0.380
